# A highly divergent *Wolbachia* with a tiny genome in an insect-parasitic tylenchid nematode

**DOI:** 10.1098/rspb.2022.1518

**Published:** 2022-09-28

**Authors:** Jan P. Dudzic, Caitlin I. Curtis, Brent E. Gowen, Steve J. Perlman

**Affiliations:** Department of Biology, University of Victoria, Victoria, British Columbia, Canada

**Keywords:** genome reduction, nematodes, parasitism, *Spelobia*, symbiosis, *Wolbachia*

## Abstract

*Wolbachia* symbionts are the most successful host-associated microbes on the planet, infecting arthropods and nematodes. Their role in nematodes is particularly enigmatic, with filarial nematode species either 100% infected and dependent on symbionts for reproduction and development, or not at all infected. We have discovered a highly divergent strain of *Wolbachia* in an insect-parasitic tylenchid nematode, *Howardula* sp., in a nematode clade that has not previously been known to harbour *Wolbachia*. While this nematode is 100% infected with *Wolbachia*, we did not detect it in related species. We sequenced the *Howardula* symbiont (*w*How) genome and found that it is highly reduced, comprising only 550 kilobase pairs of DNA, approximately 35% smaller than the smallest *Wolbachia* nematode symbiont genomes. The *w*How genome is a subset of all other *Wolbachia* genomes and has not acquired any new genetic information. While it has lost many genes, including genes involved in cell wall synthesis and cell division, it has retained the entire haem biosynthesis pathway, suggesting that haem supplementation is critical. *w*How provides key insights into our understanding of what are the lower limits of *Wolbachia* cells, as well as the role of *Wolbachia* symbionts in the biology and convergent evolution of diverse parasitic nematodes.

## 1. Introduction

Bacteria in the genus *Wolbachia* are the most abundant host-associated microbes on the planet, successfully infecting two old and hyperdiverse groups of invertebrates—arthropods and nematodes [1–3]. Although these symbionts and how they affect their hosts are highly variable, they are united by highly efficient maternal transmission and a strong and broad affinity for germline tissue [4–7].

*Wolbachia* is especially abundant in arthropods, estimated to infect approximately 40% of terrestrial species [8]. One of the major reasons for this enormous host range is that despite being primarily maternally transmitted over short (i.e. ecological) timescales, new arthropod hosts are repeatedly colonized over longer (i.e. evolutionary) timescales, through mechanisms that are currently not understood [9]. In addition, most arthropod-infecting *Wolbachia* strains are facultative in their hosts, meaning that their hosts can survive and reproduce without them (although the converse is not true, as *Wolbachia* cannot live without their hosts). These facultative strains can affect their hosts in diverse ways, such as providing protection against pathogenic viruses [10,11] or manipulating their hosts' reproduction in order to increase the frequency of infected females (i.e. the transmitting host) [12–14], for example by causing mating incompatibilities between infected males and uninfected females. The combination of pathogen protection and mating incompatibility has generated a great deal of recent interest in using *Wolbachia* to control pests and disease vectors [15–17]. Yet not all arthropod *Wolbachia* strains are facultative in their hosts. For example, *Wolbachia* is an obligate essential symbiont of bedbugs, providing B vitamins that are absent from the bedbug's blood diet [18]; bedbugs that have had their *Wolbachia* removed via antibiotic treatment have severely impaired development and reproduction.

Patterns of *Wolbachia* infection in nematodes are markedly different from those in arthropods [3,19,20]. First, the range of nematodes that host *Wolbachia* is much more restricted. Almost all known nematode hosts are filarial nematodes, which are parasites that require a blood-feeding arthropod and a vertebrate (sometimes human) host to complete their life cycle. Second, all *Wolbachia* strains that infect filarial nematodes are obligate; hosts that are cleared of infection are unable to successfully develop and reproduce [21,22]. However, why *Wolbachia* is essential to filarial nematodes is still unknown, despite intensive study. One leading hypothesis is that it provides them with essential nutrients, similar to bedbugs. However, there is still limited evidence for this hypothesis, probably owing to the fact that it is very challenging to manipulate filarial nematodes inside their hosts. In parallel, researchers have used comparative genomics to look for biosynthetic pathways that are conserved across diverse nematode *Wolbachia* genomes, and that may give clues as to key metabolites that *Wolbachia* might provide, such as haem [23–27]. Interestingly, a number of filarial nematode species have independently lost *Wolbachia* without gaining new symbionts, genes or ecologies, making *Wolbachia*'s essentiality even more mysterious [28–30].

Unlike terrestrial arthropods, nematodes (other than filarial nematodes) have been little surveyed for *Wolbachia*, or for bacterial symbionts in general. Thus far, only two non-filarial nematodes, the plant-parasitic tylenchids *Radopholus similis* [31] and *Pratylenchus penetrans* [32] have been found to host *Wolbachia*, and virtually nothing is known about either of these infections [33,34]. It would thus be highly informative to survey a much broader diversity of nematodes. This would also help solve the mystery of whether the symbiosis originated in arthropods or in nematodes [32]. To this end, in this study, we report the discovery of a highly divergent strain of *Wolbachia* in a tylenchid nematode parasite of flies that has the hallmarks of an obligate symbiosis. All worms that we screened are infected with this symbiont. We sequenced its genome and found that it is the smallest *Wolbachia* genome by far, at approximately 550 kb, approximately 35% smaller than the previous smallest published *Wolbachia* genomes [25], and representing an intriguing new model for understanding *Wolbachia*-nematode interactions and evolution.

## 2. Results

### (a) A novel *Wolbachia* in a parasitic nematode infecting *Spelobia* flies

**Figure 1.**
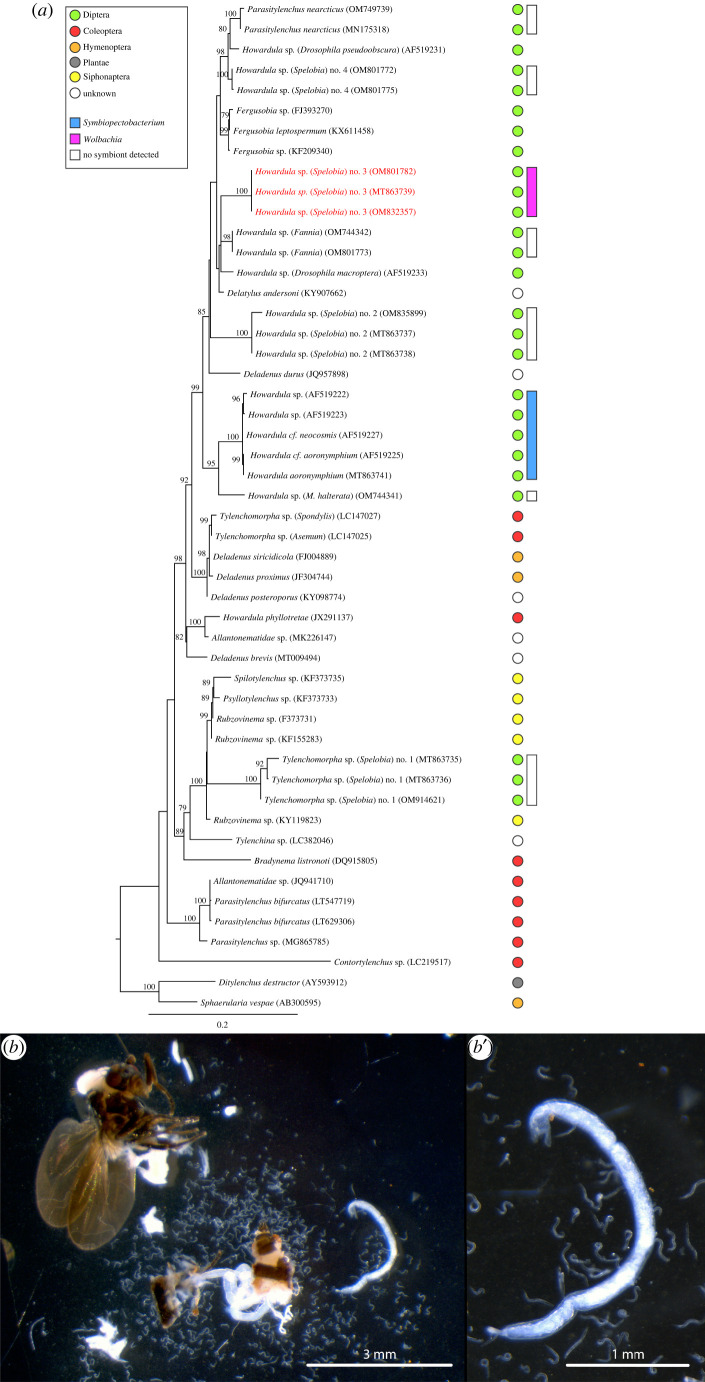
(*a*) Phylogenetic analysis of nematode 18S rRNA sequences, using maximum likelihood, and implemented with IQ-Tree, and with 1000 bootstrap replicates; bootstrap values ≥65 are indicated. The best-fit model calculated by ModelFinder was TIM3 + F + I+G4. The scale bar indicates the distance in substitutions per nucleotide. Coloured circles next to the nematode species indicate the type of host that this nematode infects: with flies (order Diptera) in green, beetles (Coleoptera) in red, wasps and allies (Hymenoptera) in orange, fleas (Siphonaptera) in yellow, plants in grey, and unknown hosts in white. Pink or blue coloured bars show nematodes that harbour *Wolbachia* or *Symbiopectobacterium* symbionts, respectively. (*b*) Dark-field micrograph of a dissected *Spelobia* sp*.* infected with nematodes. The dissection reveals an approximately 3 mm long motherworm (right side) as well as a high number of juveniles with a length of roughly 0.25 mm. (*b′*) Magnification of the motherworm. (Online version in colour.)

Sphaerocerid flies, primarily from the genus *Spelobia*, were abundant at our mushroom baits. While less than 5% of sphaerocerids were infected by nematodes, there were four morphologically distinct tylenchid nematodes in these samples, infecting *Spelobia ordinaria*, *Spelobia quinata*, an unidentified *Spelobia species* and *Minilimosina fungicola* (electronic supplementary material, table S1). 18S rRNA sequencing confirmed that these four nematodes are distinct (figure 1*a*). These nematodes are all undescribed species, and may also represent new genera. One of these nematode species (species no. 1) is most closely related to *Rubzovinema* nematode parasites of fleas [35], while the other three (*Howardula* species nos 2, 3 and 4) are found in a clade of nematode parasites that infects diverse flies. (Note that *Howardula* is a polyphyletic group of nematodes that infects a wide range of insects, and in major need of taxonomic revision [35,36].)

16S rRNA screening of nematodes for bacterial symbionts revealed the presence of a divergent *Wolbachia* in *Howardula* species no. 3, a nematode with distinctively long and thin motherworms (figure 1*b*,*b*׳). We refer to this *Wolbachia* strain as *w*How. Phylogenetic analysis of the *Wolbachia* 16S rRNA sequence shows a highly divergent taxon with no affiliation to any of the already known *Wolbachia* supergroups (electronic supplementary material, figure S1). Using primers targeting *w*How 16S rRNA, we found that 100% of *Howardula* sp. no. 3 individuals harboured *Wolbachia* (*n* = 32; electronic supplementary material, table S1). We barcoded all wild-caught flies infected with *Howardula* sp. no. 3; all but one were an unidentified *Spelobia* sp. (greater than 99.5% similar to Genbank accession MT863700), while the other was *S. quinata* [37]. To confirm that the *Wolbachia* symbiont is restricted to the nematode, we screened 26 uninfected *Spelobia* sp. hosts of *Howardula* sp. no. 3 (greater than 99.5% similar to Genbank accession MT863700) collected at the same baits; none were infected with *Wolbachia*. We did not recover *Wolbachia* from any of the other sphaerocerid-parasitic nematode samples (electronic supplementary material, table S1). We also screened nematodes from four additional species in the fly parasite clade; two parasites of *Drosophila* (*Howardula aoronymphium* and *Parasitylenchus nearcticus*), and nematodes infecting *Megaselia halterata* (Diptera: Phoridae) and *Fannia* sp. (Diptera: Fanniidae) (electronic supplementary material, table S1). We were unable to detect *Wolbachia* in any of these nematodes, except for samples infecting *Fannia* and *Megaselia*; these are probably insect *Wolbachia*, as their 16S rRNA, wsp and coxA sequences are greater than 99.5% similar to symbionts in arthropod-infecting supergroups A and B.

### (b) *Wolbachia* symbiont of *Howardula* nematode is highly divergent and has a tiny genome

**Figure 2.**
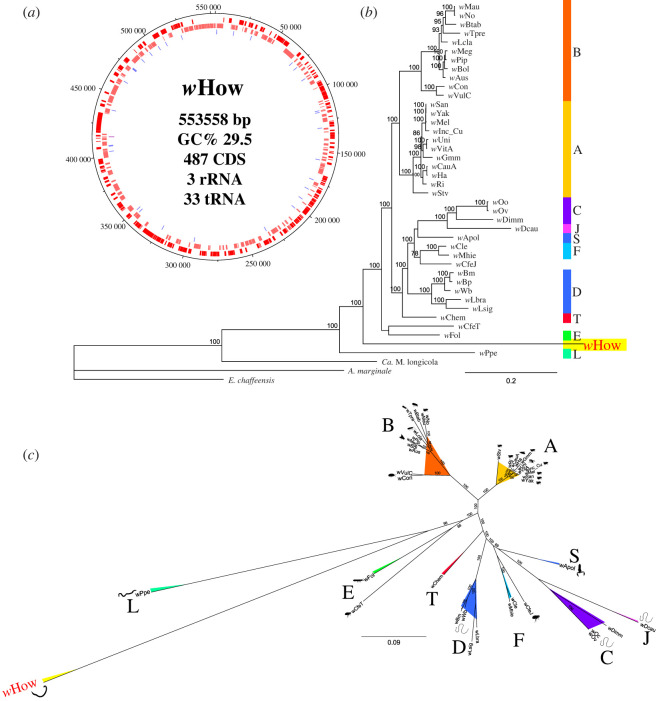
(*a*) Circular overview of the 553,558 bp *w*How genome. Red bars indicate the 487 identified CDS (outer ring = positive-sense, inner ring = negative-sense). Blue and pink bars indicate identified transfer RNAs (tRNAs) [33] and ribosomal RNAs (rRNAs) [3], respectively. The GC content is 29.5%. (*b* + *c*) Phylogenomic analysis of *Wolbachia*, using maximum likelihood, and implemented with IQ-Tree, and with 1000 bootstrap replicates; bootstrap values ≥65 are indicated. The scale bar indicates the distance in substitutions per nucleotide. Colours correspond to *Wolbachia* supergroups. (*b*) Rooted phylogenomic tree based on 132 single-copy orthologous genes found in all used *Wolbachia* genomes (*n* = 40) as well as the close relatives *Candidatus* Mesenet longicola, *Anaplasma marginale* and *Ehrlichia chaffeensis*. The best-fit model calculated by ModelFinder was JTT + F+I + G4. (*c*) Unrooted tree based on 145 single-copy orthologous genes that are conserved in *Wolbachia* genomes (*n* = 40). The best-fit model calculated by ModelFinder was HIVb + F+I + G4. Icons depicting *Wolbachia* hosts are from Lefoulon *et al.* [25], with a new icon for *w*How host. (Online version in colour.).

Whole-genome sequencing using Illumina and Oxford's Nanopore MinION technology led to the assembly of a circular 553 kb *w*How genome, containing 487 coding sequences (CDS), seven pseudogenes and with a BUSCO completeness score of 86.86% (figure 2*a*, table 1; Genbank accession CP092368.1). Its per cent GC content is 29.5, which lies within the range (28–36%) of *Wolbachia* symbionts of filarial nematodes [25]. Circularization of the *w*How chromosome was confirmed by polymerase chain reaction (PCR) and Sanger sequencing. The genome size of *w*How is approximately 35% smaller than the previously published smallest *Wolbachia* genomes with approximately 863 kb of *w*Ctub and *w*Dcau, symbionts of filarial nematodes [25]. The genome does not contain any identifiable mobile elements, ankyrin or phage-related genes (table 1).

**Table 1.** Genomic characteristics of the *w*How *Wolbachia* strain.

**strain**	***w*How**
host	*Howardula* sp*.* no*.* 3
genome size (bp)	553 558
proteins/hypothetical	487/9
tRNA genes	33
rRNA genes	3
% GC	29.5
% completeness (Rickettsiales)	86.86
transposases	0
ankyrin genes	0
phage-related genes	0
pseudogenes (Prokka/Pseudofinder)	5/7
signal peptides	12
plasmid	no evidence
coding density %	86

For a detailed phylogenetic analysis, we identified a total of 132 single-copy orthologous genes from three close relatives of *Wolbachia*: *Candidatus* Mesenet longicola, *Anaplasma marginale* and *Ehrlichia chaffeensis* as well as from across 39 published *Wolbachia* genomes and *w*How (figure 2*b*; electronic supplementary material, table S2). This confirmed our 16S rRNA phylogenetic analyses and places *w*How in a novel, divergent clade, and which probably represents a new *Wolbachia* supergroup. We obtained similar results upon performing phylogenetic analyses using only *Wolbachia* genomes (i.e. without *Ca*. M. longicola, *A. marginale* and *E. chaffeensis*), and a total of 145 single-copy orthologous genes across all 40 *Wolbachia* genomes (figure 2*c*). We also calculated average nucleotide identity; this confirms that *w*How is highly divergent, with 71–74% similarity to other *Wolbachia* genomes (electronic supplementary material, table S3).

### (c) Comparative genomics of *w*How

We compared the content of the *w*How genome with 45 representative *Wolbachia* genomes, using anvi'o (electronic supplementary material, figure S2, table S2). There was strong concordance between *Wolbachia* supergroup phylogenetic relationships and gene content, with the exception of symbionts in supergroup D, which were split into two groups, reflecting their different genome sizes. From our pangenome analysis, we identify a core *Wolbachia* genome of 347 gene clusters that is present in all *Wolbachia* genomes (electronic supplementary material, data file S1). Removing *w*How from this analysis increased the number of conserved *Wolbachia* gene clusters by approximately 20%. We were therefore intrigued by what genes and gene pathways are retained or lost in *w*How, as these may reveal features that are critical for *Wolbachia* function, focusing in particular on *Wolbachia* that infect nematodes [25].

To our surprise, *w*How has completely lost most genes involved in the synthesis of a bacterial cell wall, belonging to the KEGG pathways for peptidoglycan biosynthesis (KO:00550) and lysine biosynthesis (KO:00300) (electronic supplementary material, figure S3). While these genes, necessary for the production and organization of peptidoglycan, are highly conserved in all other *Wolbachia* [38], *w*How seemingly has lost the ability to synthesize a bacterial cell wall or sacculus (electronic supplementary material, figure S4), which is commonly observed in symbiotic bacteria with minimal genomes [39]. Furthermore, we found a significant loss of genes involved in the cell cycle pathway in *w*How compared to other *Wolbachia* (electronic supplementary material, figure S3), including the cell division gene *ftsZ*, which, notably, is used in *Wolbachia* multilocus sequence typing (MLST) [40]; *hcpA*, another MLST gene, is also missing from *w*How. *w*How also has lost a number of genes involved in DNA recombination and repair, including the entire mismatch repair pathway (*mutS*, *mutL*, *mutH*), the recA-associated genes *topB, helD* and *priA*, as well as the *lexA*-associated genes *uvrA, uvrB*, *dinS* and *ftsK* (electronic supplementary material, table S4).

Regarding metabolic pathways that might indicate a nutritional role for *Wolbachia*, we found that *w*How does not have any genes involved in the pyridoxine (vitamin B6), biotin (vitamin B7), and folate (vitamin B9) metabolism pathways, indicating that it does not provide its nematode host with these compounds. Similar to *Wolbachia* in plant-parasitic nematodes, *w*How has almost completely lost riboflavin metabolism genes (vitamin B2)*,* except for *ribB*. By contrast, we were able to confirm the presence of thiamine metabolism (vitamin B1) in *w*How, similar to nematode-associated *Wolbachia* in supergroups C, J and L. The *de novo* synthesis pathways for fatty acids, pyrimidines, and purines are also complete, except for two missing genes (*purB*, *dgt*) in the latter pathway, indicating their importance for either *Wolbachia* or its host. *w*How has also retained a complete haem metabolism pathway, except for the haem storage protein bacterioferritin (*bfr*), which is also missing in *Wolbachia* from supergroups C and J.

The retention of specific transporters can also provide valuable clues into how symbionts interact with their hosts. *Wolbachia* in supergroups A and B have transporters for haem, phosphate, lipoproteins, zinc, biotin, iron and phospholipids (electronic supplementary material, figure S3). *Wolbachia* from supergroups C and D have only lost a few individual genes in these pathways, which is unlikely to reduce their ability to transport those molecules. Interestingly, while symbionts from supergroup J show loss in the ability to transport biotin, iron and phospholipids, transport capability is even further reduced in *w*How, where only genes for haem, phosphate and zinc transporters are retained. Relative to other *Wolbachia*, *w*How has also lost a large number of genes involved in the production of glycerophospholipids, which play essential roles as membrane constituents or in the formation of specialized membrane domains (electronic supplementary material, figure S3). Finally, most *Wolbachia* genomes contain a broad range of secretion systems (SS; type I, II and type IV), although symbionts in supergroups C, J and L have lost the type II secretion system (*gspD*). By contrast, *wHow* has not only lost the type II SS, but also the type I and type IV SS (electronic supplementary material, figure S5); however, the Sec translocase/signal recognition particle pathway and the twin-arginine targeting systems are still retained, suggesting at least residual abilities to interact with the host. Accordingly, the number of *w*How proteins containing a signal peptide is remarkably small, at only 12 (electronic supplementary material, table S5).

### (d) Microscopy and localization of *w*How

**Figure 3.**
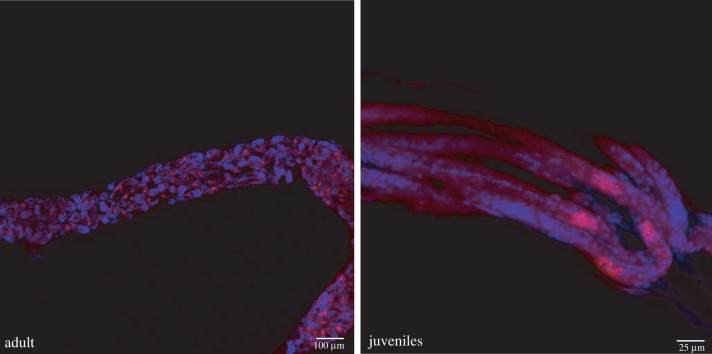
Localization of *w*How inside nematodes via 16S rRNA fluorescence *in situ* hybridization (FISH). Left side: image of an adult motherworm containing multiple juveniles. FISH staining is confined to a small area within each juvenile. Right side: higher magnification of juveniles. Only a handful of cells are positive for *w*How in each juvenile. The stained area is confined to either the apical or caudal side of the juveniles. Blue, DAPI; red, 16S rRNA FISH probe. (Online version in colour.)

**Figure 4.**
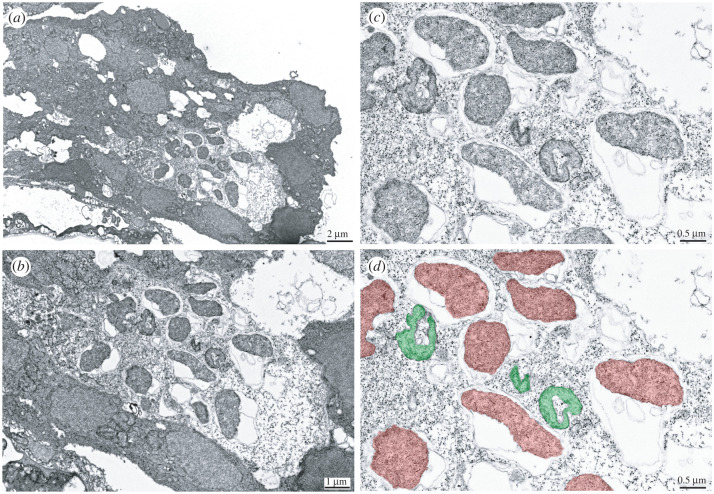
Shape and localization of *w*How via TEM. (*a*) low magnification image of a *Howardula* nematode motherworm containing *w*How. (*b*) higher magnification of the same area within the section highlighting the irregular shapes of *w*How. (*c*) higher magnification showing the lack of typical bacterial cell walls present in these bacteria. (*d*) is a falsely coloured version of (*c*) to highlight *w*How individuals (coloured red). Mitochondria present in the section have been coloured green. (Online version in colour.)

In order to localize *w*How inside its nematode host, we designed a *w*How-specific 16S rRNA probe for fluorescence *in situ* hybridization (FISH) and visualized samples via confocal laser scanning microscopy. *w*How was not found throughout the whole body of nematodes, but rather in a surprisingly narrow and confined area (figure 3, left). Like *Drosophila*-parasitic *Howardula aoronymphium* [41], *Howardula* species no. 3 is viviparous, with numerous juvenile worms developing within motherworms and then being released in the fly body cavity. In mature motherworms with internally developing juveniles, we were able to detect *w*How in every single juvenile in a similar, confined area. Higher magnification revealed a small number of cells in close proximity that were positive for *w*How (figure 3, right), which might indicate specialized cells or a specialized organ harbouring *w*How. We also performed transmission electron microscopy (TEM) to investigate *w*How's appearance. We were especially interested to see whether we might detect signatures of *w*How having lost the ability to make peptidoglycan. Interestingly, in all of our samples, *w*How cells were irregularly shaped (figure 4), although since we were only able to image nematodes from one wild-caught fly, it is premature to generalize. *w*How were also found to reside in vacuoles, which is a common feature of *Wolbachia*.

## 3. Discussion

Here we report the discovery of a highly divergent and tiny-genomed lineage of *Wolbachia* infecting a new group of hosts, insect-parasitic nematodes in an undescribed *Howardula* species (order Rhabditida, suborder Tylenchina, superfamily Sphaerulariodea). *Wolbachia* infections have been reported in two other nematode lineages [3], neither of which are closely related to this one. Almost all known nematode hosts are filarial nematodes, which are in a different suborder, Spirurina. While some species of filarial nematode, such as *Loa loa*, do not harbour *Wolbachia*, those that do are 100% infected, as in *Howardula* sp. no. 3. In addition, *Wolbachia* has been found to infect two plant-parasitic nematode species, *Pr. penetrans* and *R. similis*, which are in a different superfamily, Tylenchoidea, in the Tylenchina, so very distantly related to *Howardula* sp. no. 3. It is difficult to estimate how many millions of years separate these lineages; as a comparison, the congeners *Caenorhabditis elegans* and *Caenorhabditis briggsae* are estimated to have diverged on the order of 100 Ma [42]. Virtually nothing is known about the plant-parasitic nematode *Wolbachia*, but it is facultative in *Pr*. *penetrans* [34].

The discovery of *Wolbachia* in an entomoparasitic nematode serves to remind that one should be careful to ascribe *Wolbachia* infections to the correct host. This problem is particularly pertinent with respect to parasitoids of insects and arachnids [43,44], as *Wolbachia* is highly prevalent across terrestrial arthropods. As our newly discovered nematode symbiont is so divergent from arthropod *Wolbachia*, it should not be too difficult to differentiate it; perhaps a greater concern is that identification of *Wolbachia* in entomoparasitic nematodes may be obscured by the presence of other *Wolbachia* that infect the nematode's insect host.

We found *w*How in 100% of wild *Howardula* sp. no. 3 nematodes. In combination with its extremely reduced genome, this suggests that it is essential for its host, although one must be careful not to assume this. For example, *Westeberhardia*, a recently discovered intracellular bacterial symbiont with a streamlined genome that has retained genes for building a strong insect cuticle, is found in most (approx. 80%), but not all, populations of an invasive ant, as well as its sister species [45], showing that it is not essential, despite a highly reduced genome. Demonstrating that *w*How is obligate will of course require experimental confirmation; however, this may be quite challenging, especially as we are not yet able to maintain *Howardula* sp. no. 3 in the laboratory. It is also found at a very low prevalence (approx. 1–5%) at the field site in Victoria where we repeatedly sample, and only in spring and autumn, making it difficult to study. *Howardula* sp. no. 3 was previously collected in eastern North America [46], and it will be useful to screen for *w*How across its range.

The distribution of *w*How within its worm host also suggests an intimate association, as we observed hybridization patterns that appear to correspond to highly localized regions inside juveniles developing within motherworms. It is tempting to speculate that these localized regions correspond to bacteriomes, but we are currently constrained by limited spatial resolution and challenges in obtaining field samples to confirm this.

*Wolbachia*'s narrow host range in insect-parasitic tylenchids is puzzling. From *w*How's highly reduced genome, we would have expected to find *Wolbachia* infecting related nematodes, and to cospeciate with its hosts. Yet we were not able to detect *Wolbachia* in samples from seven related fly parasitic tylenchid nematode species, except for strains from supergroups A and B that are most likely symbionts of the nematode's insect host; which is similar to a previous study that detected the same strain of *Wolbachia* in a species of thrips and a nematode of thrips [47]. One possibility is that *Wolbachia* was lost from other fly parasitic tylenchid nematodes, and perhaps was replaced by another bacterial symbiont [48]. However, as yet, we have not found evidence of any other obligate bacterial symbionts in fly parasitic tylenchids, except for a lineage of *Symbiopectobacterium* that has recently colonized *Drosophila*-parasitic *Howardula* [49], and whose genome, unlike *w*How, bears the hallmarks of a recent symbiosis, as it is large (4.5 Mb), with over a thousand pseudogenes. Another possibility is that *Wolbachia* was perhaps acquired by *Howardula* nematodes relatively recently, from an as yet unknown lineage of hosts where it has been a symbiont for a long time. For example, some species of adelgids, insects that feed on conifer sap, have recently acquired obligate nutritional symbionts that are closely related to obligate bacterial symbionts of fungi [50,51]. It is interesting that the clade of fly parasitic *Howardula* nematodes and allies appears to contain mixtures of species that are free of symbionts, along with ones that have recently acquired putatively obligate symbionts. Many insect-parasitic tylenchids have free-living life stages that feed on fungal or plant material [52–54], and we speculate that the acquisition of symbionts is associated with a loss of this free-living stage, although most nematodes in this group are so poorly studied that it is often not known if they contain a fungal or plant-feeding stage.

The *w*How genome is striking in its degree of reduction and at approximately 550 kb is by far the smallest *Wolbachia* genome described thus far, with features typical of tiny symbiont genomes, including retention of only a small number of genes, few pseudogenes and high coding density. While the genomes of facultative *Wolbachia* symbionts of arthropods, such as strains that cause cytoplasmic incompatibility, lie in the range of approximately 1–1.5 Mb, genomes of obligate *Wolbachia* symbionts of filarial nematodes are smaller, ranging from 860 kb to approximately 1.1 Mb. Up to now, the smallest reported *Wolbachia* genome, described in a recent preprint, is that of a symbiont of *Menacanthus* chewing lice, within *Wolbachia* supergroup F, a group that includes symbionts of other blood-feeding insects and some filarial nematodes, at approximately 733 kbp [55]. Another common feature of obligate symbionts is loss of genes involved in DNA replication, repair and recombination, which is thought to drive incredibly rapid rates of substitution [39,56,57]. It is notable that *w*How has lost the entire mismatch repair pathway. This may explain why it lies on such a long branch, although it would be useful to obtain sequence information from closer relatives of *w*How, in order to accurately infer rates of evolution.

The *w*How genome gives us insight into the lower limits of what defines a *Wolbachia* cell, with many genes that were previously considered core *Wolbachia* genes missing. Notably, *w*How has lost all genes involved in cell wall synthesis and cell division. These genes have also been lost in the smallest known symbiont genomes, such as *Tremblaya*, *Hodgkinia* and *Carsonella*, obligate nutritional symbionts of sap-feeding insects, and contributing to their irregular and inconsistent shapes [39], which we also see in our electron micrographs of *w*How. All other *Wolbachia* have retained at least some peptidoglycan genes and the ability to build a cell wall, with a minimum set of genes for cell elongation and division [38,58,59]. How *w*How and the other tiny-genomed symbionts complete cell division is not known. One possibility is that this role has been taken over by the host [60], but as yet, we do not have any evidence for this in the *Wolbachia-Howardula* symbiosis.

Despite being so highly divergent, the *w*How genome is a complete subset of all other *Wolbachia*, unlike the reduced genome *Wolbachia* in *Menacanthus* chewing lice, for example, which has horizontally acquired genes involved in panthotenate synthesis [55]. Interestingly, while *w*How has lost a huge number of genes, it has retained some, but not all, pathways that are also found in filarial nematode symbionts, which have been independently colonized at least three times by different lineages of *Wolbachia* [25], all of which have converged on a similar complement of genes. Thus, *w*How provides useful clues as to the role of *Wolbachia* in filarial nematodes, which surprisingly, is still largely unresolved. A number of not mutually exclusive hypotheses have been proposed, including that *Wolbachia* provides filarial nematodes with essential and limiting nutrients and/or facilitate nematode parasitism by modulating the vertebrate immune response [23,26,61]. Recent beautiful imaging studies in *Brugia* nematodes have shown that *Wolbachia* are intricately tied to germline proliferation, prompting the suggestion that they have become essential for development [4,62]. It is especially puzzling that some filarial nematode species have independently lost *Wolbachia* without acquiring new symbionts, genes, or lifestyles [63].

In this regard, it is striking that *w*How has retained all the genes required to synthesize haem, strongly suggesting that this is the key nutrient shaping obligate symbiosis between nematodes and *Wolbachia* [26,27]. Nematodes are the only animals that have lost the ability to synthesize haem [64], and as a result have evolved sophisticated strategies to scavenge haem from their environment. At least two lineages of animal-parasitic nematodes, including filarial nematodes, have independently acquired haem biosynthesis genes via lateral gene transfer [65,66], suggesting that haem is highly limiting at certain times in their life cycle; the presence of haem biosynthesis genes in the genomes of both filarial nematodes and their *Wolbachia* symbionts makes it challenging to disentangle their respective contributions to nematode haem budgets. Finally, as *w*How's nematode host only infects flies and not vertebrates, it also suggests that the interface between nematodes and arthropod hosts is a critical juncture in understanding *Wolbachia*'s function [67].

## 4. Material and methods

### (a) Fly and nematode collections, DNA extraction, barcoding and next-generation sequencing

Mushroom-feeding woodland flies are infected with diverse tylenchid nematode parasites [46,68]. In order to characterize nematode diversity and to survey nematodes for bacterial symbionts, we used store-bought *Agaricus bisporus* mushrooms as baits to collect mycophagous flies in the woods near U. Victoria campus, Victoria, British Columbia, in the summers of 2019, 2020 and 2021, focusing in particular on flies in the family Sphaeroceridae, which were especially abundant. This work was also motivated by our recent finding that *Drosophila*-parasitic *Howardula* nematodes harbour obligate *Symbiopectobacterium* symbionts [49]. We also screened *Howardula* nematode parasites (probably *Howardula husseyi* [69]), infecting a laboratory colony of *M. halterata*, a pest of mushroom houses. Flies were brought back to the laboratory and dissected. DNA was extracted from flies and nematode motherworms separately. DNA extraction methods for PCR, Sanger and next-generation sequencing are described in the electronic supplementary material, methods. Nematodes and host flies were DNA barcoded, using 18S rRNA and mitochondrial cytochrome *c* oxidase subunit I (COI), respectively (see the electronic supplementary material, table S6).

### (b) Screening for *Wolbachia*

While screening for potential bacterial symbionts by sequencing products amplified with universal 16S rRNA primers, we stumbled on a divergent 16S *Wolbachia* sequence associated with an undescribed *Howardula* species infecting mushroom-feeding flies in the genus *Spelobia* (Diptera: Sphaeroceridae), and which we refer to as *w*How. As a number of commonly used *Wolbachia*-specific primers did not produce any PCR products, including primers designed to amplify 16S rRNA [70], wsp [71], and coxA, fbpA, ftsZ, hcpA MLST primers [40], we designed a new set of primers, amplifying an approximately 340 bp fragment of the *w*How 16S rRNA gene (primer names: wol_HA-SPA F1 and wol_HA-SPA R1) (see the electronic supplementary material, table S6); sequences were confirmed by Sanger sequencing PCR products. Universal 16S rRNA screening did not reveal any other potential *Howardula* sp. no. 3 symbionts (see also [49]).

Two other nematodes, infecting *Fannia* sp., and *M. halterata*, amplified *Wolbachia* 16S rRNA sequences that upon sequencing were found to be closely related to insect *Wolbachia* (i.e. supergroups A and B); for these we also amplified and sequenced wsp and coxA, confirming that these *Wolbachia* were indeed fly and not nematode symbionts. Finally, for nematode species for which we were unable to amplify *Wolbachia*, we also used the universal 16S RNA primers 63F and 907R. A phylogenetic tree of 16S rRNA sequences was generated as described above containing sequences listed in the electronic supplementary matetial, data file S2.

### (c) Genome assembly, annotation and comparative genomic analysis

See the electronic supplementary material, methods.

### (d) Fluorescence *in situ* hybridization

We designed a 16S rRNA *w*How-specific Alexa 594-coupled FISH probe (5′ Alexa-594-GGAGTCTGGACCGTATCTCA-3′, produced by Integrated DNA Technologies Inc.). Flies were sacrificed in 70% ethanol (EtOH) and subsequently dissected in ice-cold phosphate-buffered saline (PBS). Motherworms were fixed in Carnoy's solution (six parts anhydrous ethanol, three parts chloroform, one part acetic acid) at room temperature overnight, rinsed twice in anhydrous ethanol and then stored at −20°C until further use. Prior to hybridization, samples were re-hydrated twice in 70% and twice in 50% EtOH for two minutes each before transferring them in ddH_2_O for 3 min. Motherworms were then permeabilized in PBSTx (0.3% Triton-X in PBS pH 8.0) for 2 h at room temperature. Samples were then equilibrated in hybridization buffer (5 M NaCl, 1 M Tris-HCl pH 8.0, 20% Formamide, 5% sodium dodecyl sulfate) at 46°C for 1 hour and the probe was then hybridized in the same buffer with 200 µM probe. The samples were washed in PBSTx at 48°C for 15 min twice and then in 4°C ddH2O for 5 min twice. We finally mounted the samples in ProLong Glass Antifade Mountant with NucBlue (ThermoFisher) and let them set overnight in the dark. Fluorescence confocal microscopy was performed using a confocal microscope (Nikon C2; Nikon Corp., Tokyo, Japan) and images were processed in ImageJ [72]. As negative control, we used both *Spelobia* sp*.* guts and a *Wolbachia*-free nematode species (*Howardula aoronymphium,* descended from infected *Drosophila falleni* collected in West Hartford, CT, USA, in 2006, and maintained in our laboratory since, see also [49]), both of which showed no staining with our probe.

### (e) Transmission electron microscopy

We performed TEM of an individual *Howardula* sp. no. 3 motherworm dissected from a wild-caught fly. Samples were double fixed and EMBed-812 (Epon replacement, Electron Microscopy Sciences) embedded using standard TEM methodology [73]. After initial fixation in Karnovsky's fixative, the small samples were embedded within low melt agarose (Sigma A9045) and the resulting blocks osmicated, dehydrated, infiltrated with EMBed-812, and then polymerized. TEM sections were stained with uranyl acetate and lead citrate and viewed in a Jeol JEM 1400 TEM at 80 kV. Images were captured using a Gatan SC-1000 digital camera.

## Data Availability

All data can be accessed via Dryad Digital Repository: https://doi.org/10.5061/dryad.98sf7m0ms [74]. The data are provided in the electronic supplementary material [75].
